# Dihydroergotamine Increases Histamine Brain Levels and Improves Memory in a Scopolamine-Induced Amnesia Model

**DOI:** 10.3390/ijms25073710

**Published:** 2024-03-26

**Authors:** Maricarmen Hernández-Rodríguez, Elvia Mera Jiménez, María Inés Nicolás-Vázquez, Rene Miranda-Ruvalcaba

**Affiliations:** 1 Laboratorio de Cultivo Celular, Neurofarmacología y Conducta, Escuela Superior de Medicina, Instituto Politécnico Nacional, Plan de San Luis y Díaz Mirón s/n, Ciudad de México 11340, Mexico; 2 Departamento de Ciencias Químicas, Facultad de Estudios Superiores Cuautitlán Campo 1, Universidad Nacional Autónoma de México, Avenida 1o de Mayo s/n, Colonia Santa María las Torres, Cuautitlán Izcalli 54740, Mexico; nicovain@yahoo.com.mx (M.I.N.-V.); mirruv@yahoo.com.mx (R.M.-R.)

**Keywords:** histamine, dihydroergotamine, Morris water maze, novel object recognition

## Abstract

The beneficial effects of increasing histamine levels on memory have acquired special interest due to their applicability to psychiatric conditions that cause memory impairments. In addition, by employing drug repurposing approaches, it was demonstrated that dihydroergotamine (DHE), an FDA drug approved to treat migraines, inhibits Histamine N Methyl Transferase (HNMT), the enzyme responsible for the inactivation of histamine in the brain. For this reason, in the present work, the effect of DHE on histamine levels in the hippocampus and its effects on memory was evaluated, employing the scopolamine-induced amnesia model, the Novel Object Recognition (NOR) paradigm, and the Morris Water Maze (MWM). Furthermore, the role of histamine 1 receptor (H1R) and histamine 2 receptor (H2R) antagonists in the improvement in memory produced by DHE in the scopolamine-induced amnesia model was evaluated. Results showed that the rats that received DHE (10 mg/kg, i.p.) showed increased histamine levels in the hippocampus after 1 h of administration but not after 5 h. In behavioral assays, it was shown that DHE (1 mg/kg, i.p.) administered 20 min before the training reversed the memory impairment produced by the administration of scopolamine (2 mg/kg, i.p.) immediately after the training in the NOR paradigm and MWM. Additionally, the effects in memory produced by DHE were blocked by pre-treatment with pyrilamine (20 mg/kg, i.p.) administered 30 min before the training in the NOR paradigm and MWM. These findings allow us to demonstrate that DHE improves memory in a scopolamine-induced amnesia model through increasing histamine levels at the hippocampus due to its activity as an HNMT inhibitor.

## 1. Introduction

Histamine is a biogenic amine that acts as a neurotransmitter to regulate several physiological functions in the brain, including wakefulness, feeding, energy intake, and memory, among others [1]. In the brain, histamine is produced by histaminergic neurons located in the tuberomamilar nucleus (TMN) by the enzyme histidine decarboxylase (HDC), which employs histidine as a substrate. Histamine exerts its effects by interacting with four types of histamine receptors: H1R, H2R, H3R, and H4R, which are protein G-coupled receptors [2]. H1R and H2R potentiate excitatory inputs while H3R downregulates histamine synthesis and release as well as the release of other neurotransmitters [3]. In contrast, although there is debate about the expression of H4R in the brain, it has been demonstrated that H4R regulates cytokine release by microglial cells [4]. 

The regulation of histamine levels in the synaptic cleft occurs predominantly through the action of the enzyme Histamine N Methyl Transferase (HNMT), located in astrocytes, which inactivates histamine to *t*-methylhistamine [5]. Thus, increased histamine levels in the brain can be achieved by inhibiting HNMT, as was demonstrated by metoprine, an HNMT inhibitor [6]. 

Furthermore, the beneficial effects of increasing histamine levels in the brain were first described in 1986 by de Almeida and Izquierdo, showing that intra-cerebrovascular (i.c.v.) administration of 1 or 10 ng of histamine immediately after the training facilitated retention of step-down inhibitory avoidance tests in rats [7]. Interestingly, histamine infusion into the hippocampus of Wistar rats enhances retention in the inhibitory avoidance task in rats in a dose-dependent manner, and the effect was mimicked by hippocampal infusion with SKF-91844, which is an HNMT inhibitor [8]. In addition, the blockade of H1R and H2R receptors impaired memory retention in a novel object recognition (NOR) task when it was infused 30–120 min after training [9]. Furthermore, Prast H et al., 1996 showed that i.c.v. administration of histamine or histidine improved short-term recognition memory in rats. In contrast, depletion of neuronal histamine by alpha-fluoromethylhistidine (FMH) produces amnesia in rodents [10]. In addition, the effects of histamine on spatial memory have been demonstrated. Several reports have pointed out that both histamine and histidine ameliorate spatial memory deficits induced by aging, dorsal hippocampal lesions, and scopolamine, as determined by passive and active avoidance tasks and an eight-arm radial maze test for rats [11,12,13], thus highlighting the effects of the histaminergic system in memory processes. 

Indeed, the effects of metoprine on cognitive performance have been demonstrated in a mouse model of scopolamine-induced amnesia [14]. In this sense, the study of effects on memory of HNMT inhibitors is of special interest. 

**Figure 1:**
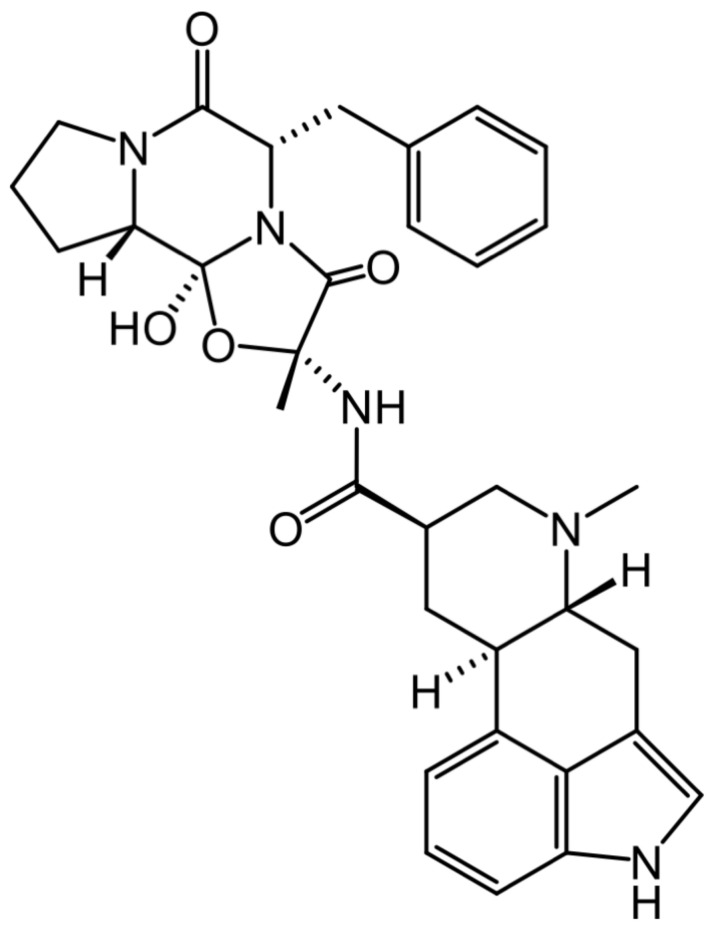
Chemical structure of dihydroergotamine (DHE).

Drug repurposing approaches employing computational and in vitro studies allowed us to demonstrate that dihydroergotamine (DHE, Figure 1), an FDA drug approved to treat migraines, inhibits HNMT [15]. For this reason, in the present work, the effect of DHE on histamine levels in the hippocampus and its effects on memory were evaluated, employing the scopolamine-induced amnesia model. 

## 2. Results

### 2.1. DHE Increases Histamine Levels in the Hippocampus

**Figure 2:**
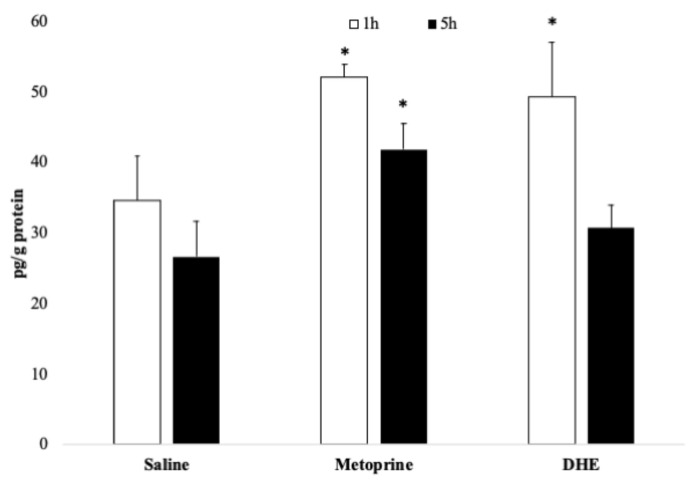
Histamine levels in the hippocampus of Wistar rats treated with metoprine and DHE. Rats were administered intraperitoneally with saline solution (2 mL/kg), metoprine (10 mg/kg), or DHE (1 mg/kg). After 1 and 5 h, rats were sacrificed and the brain histamine levels were determined employing a colorimetric competitive ELISA kit. The concentration of histamine in the samples was determined by comparing the O.D. at 450 nm with the standard curve. Data are expressed as Mean ± SEM, n = 3, and statistical analysis by one-way ANOVA (* *p* < 0.05 versus saline 1 h). For between-group comparisons, Tukey’s test was used as a post-hoc test.

Histamine levels in the hippocampus of Wistar rats after 1 and 5 h of administration of saline solution (2 mL/kg), metoprine (10 mg/kg), or DHE (1 mg/kg), by i.p. routes were determined. As can be seen in Figure 2, the treatment groups displayed increased histamine levels after 1 h of administration in comparison with the control (*p* < 0.5). Interestingly, DHE induced an increase in histamine levels in the hippocampus similar to metoprine. However, histamine levels remain elevated only in the hippocampus of rats treated with metoprine after 5 h, as was previously reported [6], but not in the rats that received DHE. 

### 2.2. DHE Improves Memory Recognition in the Scopolamine-Induced Amnesia Model

**Figure 3:**
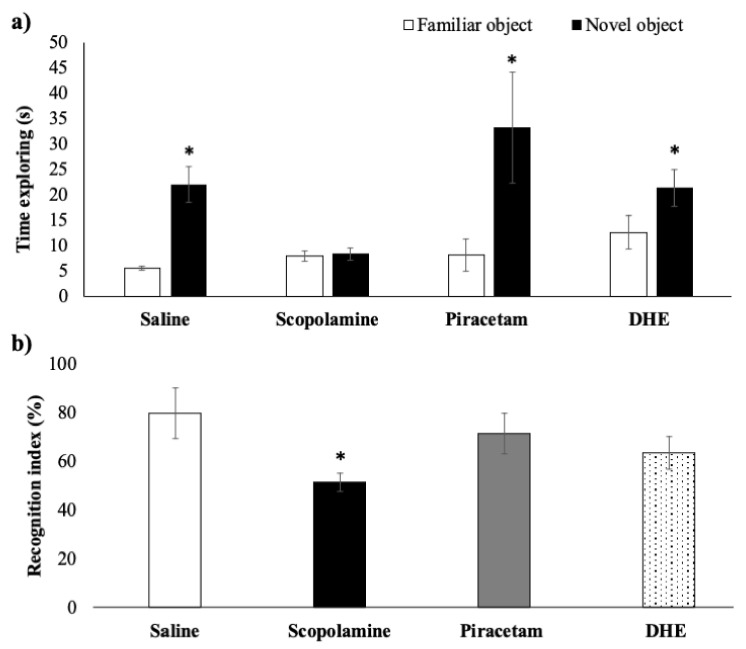
Effects of DHE in a scopolamine-induced amnesia model on recognition memory evaluated by the NOR paradigm. (**a**) In trials separated by 180 min, the rats from all groups, except for the scopolamine group, spent significantly more time exploring the novel object relative to the familiar object. Data are expressed as Mean ± SEM, n = 10. Statistical analysis was performed using paired Student *t*-test, * *p* < 0.05 were considered significant. (**b**) Graph plot for the recognition index. The recognition index is similar for all the groups except for the scopolamine group, which showed the lowest value. Data are expressed as Mean ± SEM, n = 10. Statistical analyses were performed by one-way ANOVA * *p* < 0.05. For between-group comparisons, Tukey’s test was used as a post-hoc test.

As can be seen in Figure 3a, administration of 2 mg/kg (i.p.) scopolamine immediately after the training session significantly impaired remembering the exposition to a familiar object in the scopolamine group in comparison with the saline group. This fact is evidenced by a lower recognition index of the scopolamine group in comparison with the saline group (Figure 3b). Interestingly, in the piracetam group and DHE group, the effect produced by scopolamine was reversed, thus the rats spent more time exploring the novel object than exploring the familiar object (*p* < 0.05). 

### 2.3. DHE Improves Spatial Learning and Memory in the Scopolamine-Induced Amnesia Model 

**Figure 4:**
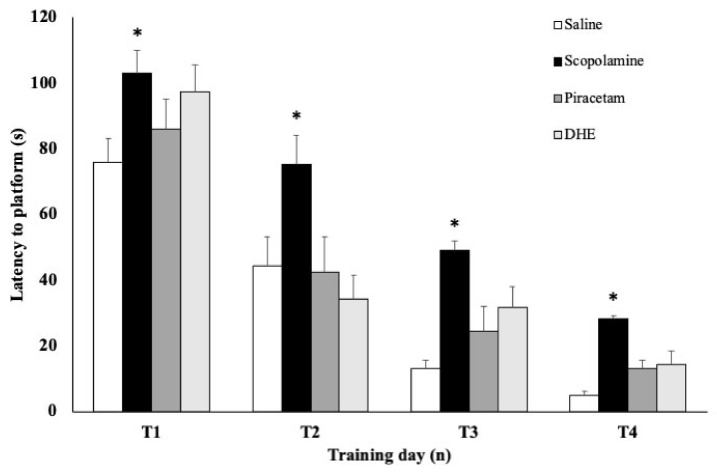
Effects of DHE in a scopolamine-induced amnesia model on escape latency during the training. This figure shows a significant increase in escape latency in the scopolamine group in comparison with the saline group. The impairment in learning was reversed by treatment with piracetam 30 mg/kg i.p. or DHE 1 mg/kg i.p. in the corresponding groups (the piracetam group and the DHE group). Values are presented as means ± SEM, n = 10. Statistical analyses were performed by one-way ANOVA * *p* < 0.05. For between-group comparisons, Tukey’s test was used as a post-hoc test.

Acquisition of the place task is represented by mean escape latencies on the four training trials. As shown in Figure 4, there is a decrease in the escape latency in all the groups on T2, T3, and T4, as compared to T1. However, the administration of scopolamine resulted in an increase in escape latency in the scopolamine group in comparison with the saline group (*p* < 0.05) during all the training days. Interestingly, treatment with piracetam 30 mg/kg i.p., a known nootropic drug, administered 20 min before the training session reverted the impairment produced by the administration of scopolamine, evidenced by similar escape latency times to the saline group (Figure 4). A similar effect was obtained with DHE (Figure 4).

**Figure 5:**
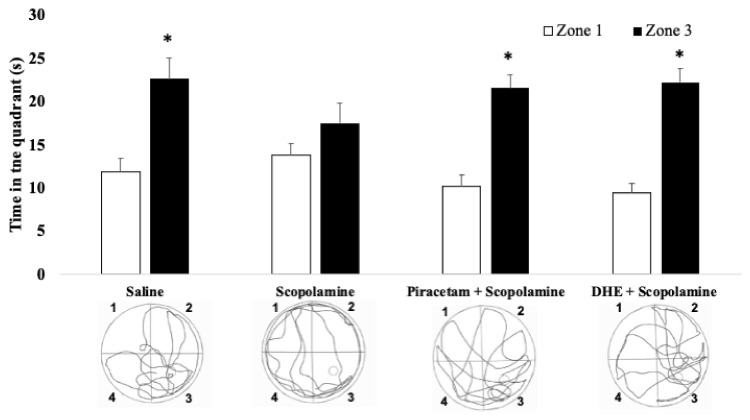
Effects of DHE in a scopolamine-induced amnesia model on path swim during the trial day. This figure shows a significantly lower time exploring quadrant 3, where the platform was located, versus the opposite quadrant 1, when the hidden platform was removed in the scopolamine group in comparison with the saline group. The impairment in remembering was reversed by treatment with piracetam 30 mg/kg i.p. or DHE 1 mg/kg i.p. in the corresponding groups (the piracetam group and the DHE group). Data are expressed as Mean ± SEM, n = 10. Statistical analysis was performed using paired Student *t*-tests, * *p* < 0.05 were considered significant A representative path swim diagram for each treatment group is shown.

**Figure 6:**
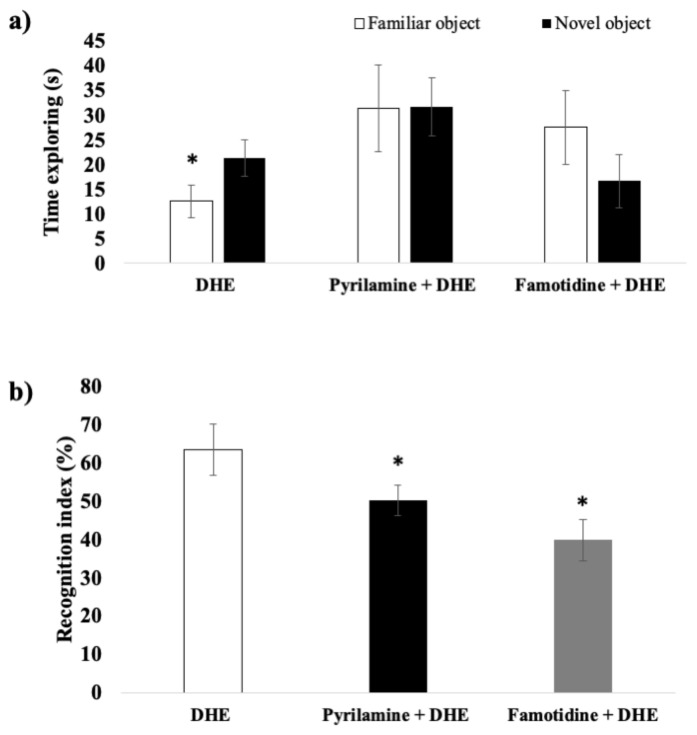
Effects of the blockade of H1R or H2R on the effects produced by DHE in a scopolamine-induced amnesia model evaluated by the NOR paradigm. (**a**) In trials separated by 180 min, the rats from all the groups, except for the scopolamine group, spent significantly more time exploring the novel object relative to the familiar object. Data are expressed as Mean ± SEM, n = 10. Statistical analysis was performed using paired Student *t*-tests, * *p* < 0.05 were considered significant. (**b**) Graph plot for the recognition index. The recognition index is similar for all the groups except for the scopolamine group, which showed the lowest value. Data are expressed as Mean ± SEM, n = 10. Statistical analyses were performed by one-way ANOVA * *p* < 0.05. For between-group comparisons, Tukey’s test was used as a post-hoc test.

In addition, as can be seen in Figure 5, during the trial day, the saline group spent more time in the target quadrant than the opposite quadrant (*p* < 0.05). In contrast, the administration of scopolamine immediately after training affects memory, as is shown in the scopolamine group, which does not show statistically significant differences in time spent in the target quadrant and the opposite quadrant (Figure 6). Interestingly, the piracetam group and the DHE group, which received treatment with piracetam 30 mg/kg i.p. or DHE 1 mg/kg i.p., respectively, reverted the impairment produced by the administration of scopolamine, evidenced by the rats spending more time in the target quadrant than the opposite quadrant (*p* < 0.05), similar to the saline group.

### 2.4. H1R and H2R Antagonists Blockade the Improvement in Recognition Memory Produced by DHE in the Scopolamine-Induced Amnesia Model

To determine the contribution of increased histamine levels in the brain on the effects on memory produced by DHE, pre-treatment with pyrilamine (an H1R antagonist) or famotidine (an H2R antagonist) was evaluated. As can be seen in Figure 6a, pre-treatment with pyrilamine (20 mg/kg, i.p.) or famotidine (10 mg/kg, i.p.) caused a reversion of the improvement in the recognition memory of DHE in the scopolamine-induced amnesia model in the Pyrilamine + DHE group and the Famotidine + DHE group, respectively. This is evidenced by the rats exploring both the novel object and the familiar object for similar periods of time (Figure 6a), and lower discrimination indices for the Pyrilamine + DHE group and the Famotidine + DHE group than the DHE group (*p* < 0.05) (Figure 6b).

### 2.5. H1R Antagonist Blockades the Improvement in Spatial Learning and Memory Produced by DHE in the Scopolamine-Induced Amnesia Model 

**Figure 7:**
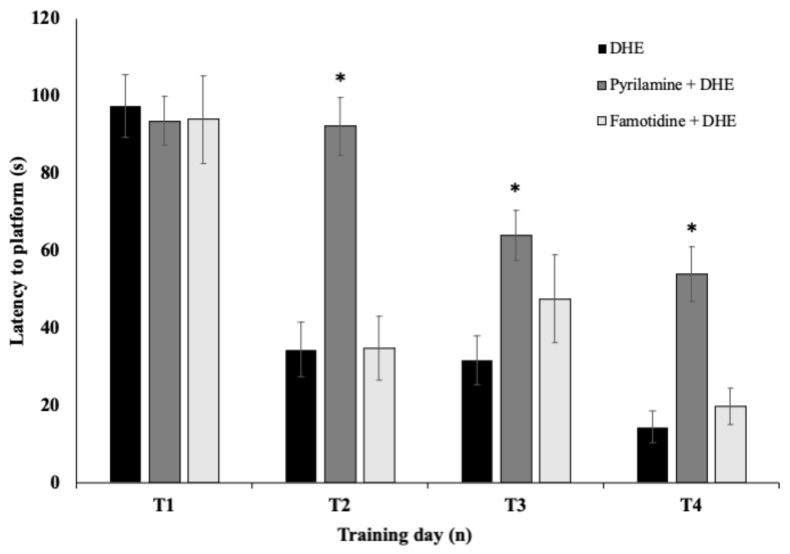
Effects of the blockade of H1R or H2R on the effects produced by DHE in a scopolamine-induced amnesia model in escape latency during the training. This figure shows a significant increase in escape latency in the Pyrilamine + DHE group, which received pre-treatment with pyrilamine 20 mg/kg i.p., in comparison with the DHE group. In contrast, pre-treatment with famotidine 10 mg/kg did not influence the anti-amnesic effect of DHE in the Famotidine + DHE group. Values are presented as means ± SEM, n = 10. Statistical analyses were performed by one-way ANOVA * *p* < 0.05. For between-group comparisons, Tukey’s test was used as a post-hoc test.

As can be seen in Figure 7, pre-treatment with pyrilamine (20 mg/kg, i.p.) in the pyrilamine + DHE group caused a reversion of the improvement of acquisition of the place task exhibited by DHE in the scopolamine-induced amnesia model (*p* < 0.05). In contrast, pre-treatment with famotidine in the famotidine + DHE group did not influence the anti-amnesic effect of DHE (Figure 7). 

**Figure 8:**
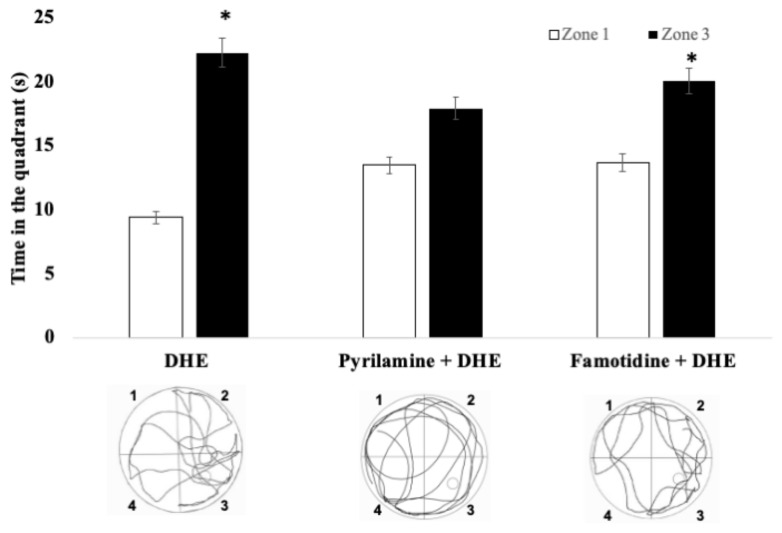
Effects of the blockade of H1R or H2R on the effects produced by DHE in a scopolamine-induced amnesia model in path swim during the trial day. Pre-treatment with pyrilamine (20 mg/kg, i.p.) or famotidine (10 mg/kg i.p.) caused a reversion of improvement in the recognition memory exhibited by DHE in the scopolamine-induced amnesia model in the Pyrilamine + DHE group and the Famotidine + DHE group, respectively. This is evidenced by the increase in the time spent in the opposite quadrant and the lowering of the time spent in the target quadrant. Data are expressed as Mean ± SEM, n = 10. Statistical analysis was performed using paired Student *t*-tests, * *p* < 0.05 were considered significant. A representative path swim diagram for each treatment group is shown.

However, as can be seen in Figure 8, during the trial day, it was shown that pyrilamine (20 mg/kg, i.p.) and famotidine (10 mg/kg i.p.) caused a reversion of improvement in the recognition memory of DHE in the scopolamine-induced amnesia model in the Pyrilamine + DHE group (*p* < 0.05). This is evidenced by the increase in the time spent in the opposite quadrant and the lowering of the time spent in the target quadrant, showing no statistically significant differences in the Pyrilamine + DHE group (Figure 8).

## 3. Discussion

Over the last forty years, it has been demonstrated that histamine is a potent modulator of memory and learning [16,17]. Experiments performed by da Silva WC et al., 2006 showed that histamine infusion into the CA1 region of the dorsal hippocampus immediately after training enhances retention of inhibitory avoidance in rats [8]. In addition to the effect of exogenous histamine application, the requirements for endogenous histamine on memory consolidation have been reported [18]. 

For these reasons, increasing histamine levels in the brain has been proposed as a promising approach to treat neurological disorders that cause memory impairments, including Alzheimer’s disease [19]. An increase in histamine levels in the brain can be achieved by inhibiting HNMT, the main enzyme that inactivates histamine in the central nervous system (CNS). Indeed, it has been demonstrated that HNMT inhibition by metoprine reverts amnesia produced by scopolamine a modified mouse passive avoidance test [14]. However, nowadays, there are few HNMT inhibitors, and most of them have a toxic adverse effect profile, including metoprine (cutaneous, gastrointestinal, and hematological toxicities) [20]. In addition, although they show HNMT inhibitory activity, some of these compounds, such as amodiaquine, do not increase brain histamine levels due to their poor blood–brain barrier (BBB) penetration [14,21]. Recently, it has been demonstrated that DHE, an FDA drug approved to treat migraines, inhibits HNMT [19]. Consequently, due to DHE being a drug employed for the treatment of neurological diseases, its safety and BBB penetration have already been corroborated. In the present study, it was demonstrated that DHE increases histamine levels in the hippocampus 1 h after i.p. administration. However, the increase in histamine levels by DHE is not maintained after 5 h of administration. The transient increase in histamine levels could be related to the DHE peak plasmatic concentration [22]. Furthermore, although it has been demonstrated that the anti-migraine effect of DHE persists for days due to tissue binding, allowing the maintenance of DHE concentrations in the picogram range [23], the concentration could be insufficient to inhibit HNMT. 

To evaluate the effects of increasing histamine levels in the hippocampus on memory, a scopolamine-induced amnesia model was employed. Scopolamine hydrobromide is a muscarinic receptor antagonist with amnestic properties that has been used for decades to induce impairment in murine performance in a variety of tasks requiring intact working and reference memory [24]. Although it has been demonstrated that chronic administration of scopolamine induces deleterious effects in the brain [25], we administered scopolamine immediately after the training due to its ability to reduce the cholinergic tone after its systemic administration by blocking muscarinic receptors in naïve animals [26]. This amnesia model allows the evaluation of several types of memory enhancers and is not only restricted to cholinergic-related compounds [27]. 

Treatments with the studied compounds were evaluated employing the NOR paradigm and the MWM assay, which are classical tasks widely used to assess memory parameters in rodents. Learning processes in both tasks involve the integrity of the hippocampus and associated regions [28]. 

The anti-amnesic effect of DHE on the NOR paradigm was evidenced by the restoration of novelty preference by spending more time on the novel object relative to the familiar object. Additionally, in the MWM assay, it was demonstrated that DHE treatment improved search errors during training trials produced by scopolamine, suggesting the amelioration of learning impairment. In addition, during the trial day, it was shown that DHE increased the time spent in the target quadrant, thus evincing the efficacy of DHE in improving memory impairment produced by scopolamine. Thus, the present study demonstrated that DHE exhibits an effect on memory similar to that previously demonstrated by metoprine [14].

In order to determine if the effects produced by DHE are related to the histaminergic system, pre-treatments with pyrilamine (H1R antagonist) or famotidine (H2R antagonist) were performed. H1R and H2R are found postsynaptically in all parts of the brain, including the cortex, hippocampus, striatum, and hypothalamus. The H1R is widely expressed in different areas of the brain, including the hippocampus where it is coupled to the Gi protein, mediating neuronal excitation through the stimulation of phospholipase C and thus the second messenger systems that lead to the release of IP_3_ and Ca^2+^ from intracellular stores where they play an important role in neuronal functionality and thus in learning and memory formation [29]. H2R is coupled to Gs and stimulates adenylyl cyclase, thereby increasing intracellular cyclic adenosine monophosphate (cAMP), which in turn activates protein kinase A (PKA) and the transcription factor cAMP response element-binding protein (CREB). The cAMP-dependent CREB function in neurons has been associated with numerous intracellular processes, such as proliferation, differentiation, survival, long-term synaptic potentiation, neurogenesis, and neuronal plasticity [30]. However, beneficial effects on memory produced by DHE in MWM were reversed only by pyrilamine (an H1R antagonist). This could be due to differences in the contribution of H1R to the memory process in comparison with H2R. Previous studies in H1R-knockout (H1R-KO) mice revealed deficits in a variety of spatial learning and memory tasks [31]. Several reports suggest that H1R deficiency impairs learning and memory in response to novel objects, although the motivation to explore novel objects was unchanged [32,33]. Moreover, spatial learning and object recognition were impaired in H1R-deficient mice. Interestingly, like H1R-deficient mice, H2R-deficient mice also show impaired object recognition but improved auditory and contextual freezing [34]. These results indicate that H2R shares some, but not all, of its behavioral effects with H1R.

Another explanation of the lack of effect of famotidine on reversing the beneficial effects of DHE on spatial memory in the present study is associated with changes in other neurotransmitter systems produced by DHE, which can contribute to their effect on memory. Piechal A. et al., 2021 demonstrated that administration of DHE to adult male Wistar Albino Glaxo rats produced changes in the concentration of monoaminergic neurotransmitters and their metabolites in the prefrontal cortex, striatum, cerebellum, medulla oblongata, and spinal cord [35]. Due to the above commentaries, it is necessary to continue with the exploration of DHE’s effects on memory in additional behavioral assays. 

## 4. Materials and Methods

### 4.1. Animals

Adult male Wistar rats weighing between 200–250 g were acquired from the animal facility of Instituto de Fisiología Celular from the Universidad Nacional Autónoma de México. The rats were kept and maintained in cages under standard conditions (12:12 h light/dark cycle, stress-free, water ad libitum, and standard diet). Prior to the experiment, the rats were allowed to acclimatize for a period of one week to reduce environmental stress. The animal procedures were conducted in accordance with the Mexican Official Standard NOM-062-ZOO-1999 [36]. 

### 4.2. Drugs

Drugs were purchased from Sigma–Aldrich (St. Louis, MO, USA). All drugs were dissolved in saline solution 0.9%. Drug concentrations were prepared in such a way that the necessary dose could be injected in a volume of 2 mL/kg by both subcutaneous intraperitoneal (i.p.) routes. Drugs were administered at the following doses: Metoprine 10 mg/kg i.p. [37], DHE 1 mg/kg i.p. [38], scopolamine 2 mg/kg i.p. [25], piracetam 30 mg/kg i.p. [14], pyrilamine 20 mg/kg i.p. [14], and famotidine 10 mg/kg i.p. [39]. 

### 4.3. Histamine Quantification in the Hippocampus

Wistar rats were intraperitoneally (i.p.) administered with the following treatments (n = 6); saline solution, metoprine (10 mg/kg), and DHE (1 mg/kg). After 1 h, three rats from each group were decapitated and the hippocampus from each group were isolated on ice. The same procedure was repeated for the remaining rats, 5 h after i.p. administration.

Hippocampal samples were rinsed with ice-cold PBS (0.01M, pH = 7.4) to remove excess hemolysis blood thoroughly. Tissue pieces were weighed, minced into small pieces, and homogenized, employing an ultrasonic cell disrupter (Tissuelyser) in PBS (4.5 mL PBS were employed for 1 g of tissue) on ice.

Histamine quantification in the hippocampus of Wistar rats was performed by employing a colorimetric Competitive ELISA kit (abcam, E.U.A., catalog number ab285333). The microtiter plate provided in this kit is pre-coated with histamine. During the reaction, histamine in the sample or standard competes with a fixed amount of histamine on the solid phase supporter for sites on the Biotinylated Detection Antibody specific to histamine. The concentration of histamine in the samples is then determined by comparing the O.D. at 450 nm with the standard curve.

### 4.4. Effects of DHE on the Scopolamine-Induced Amnesia Model 

Scopolamine is a compound widely employed to produce memory deficits by disrupting cholinergic neurotransmission [40].

Thus, scopolamine can be employed to induce an amnesia model in rodents to screen anti-amnesic drugs [41,42,43].

Due to the comments, in the present study, the scopolamine-induced amnesia model was employed to evaluate the effects of dihydroergotamine on memory deficits, employing piracetam as a positive control due to its action as a nootropic [44]. 

For this propose, forty Wistar rats were randomized into 4 groups (n = 10) to undergo behavioral assays employing the NOR paradigm (days 1 and 2) and MWM (days 3 to 7). Pre-treatments and treatments were administered for each group as follows. Saline group: receive pre-treatment with isotonic saline solution 2 mL/kg i.p. (30 min before behavioral training), treatment with saline solution 2 mL/kg i.p. (20 min before behavioral training), and saline solution 2 mL/kg i.p. immediately after the training. Scopolamine group: receive pre-treatment with isotonic saline solution 2 mL/kg i.p. (30 min before behavioral training), treatment with saline solution 2 mL/kg i.p. (20 min before behavioral training), and scopolamine 2 mg/kg i.p. immediately after the training. Piracetam group: receive pre-treatment with isotonic saline solution 2 mL/kg i.p. (30 min before behavioral training), treatment with piracetam 30 mg/kg i.p. (20 min before behavioral training), and scopolamine 2 mg/kg i.p. immediately after the training. DHE group: receive pre-treatment with isotonic saline solution 2 mL/kg i.p. (30 min before behavioral training), treatment with DHE 1 mg/kg i.p. (20 min before behavioral training), and scopolamine 2 mg/kg i.p. immediately after the training. 

#### 4.4.1. NOR Paradigm

The NOR is a memory test based on the natural propensity of rodents to explore novelty, which confers to the animals the ability to discriminate between novel and familiar entities. This paradigm is especially suited to test the effects of pharmacological interventions on learning and memory [45]. For this reason, the NOR paradigm was employed to evaluate recognition memory in the present study. For this purpose, the experimental apparatus consisted of an open field box (40 × 40 × 40 cm) made of a black acrylic material. The behavior test was conducted between 9:00 AM and 6:00 PM under dim-light illumination conditions (70 lux). The objects to be discriminated consist of a white circular cap of a culture medium bottle (familiar object) and a yellow rectangular Tupperware (new object). One day prior to the experiment, each rat was habituated to the open field box without any object for 10 min. On the experiment day, rats received pre-treatment and treatment as was previously described. After, each rat was placed in the open field for 5 min and allowed to freely explore the two identical objects (white circular cap of a culture medium bottle), receiving saline solution or scopolamine immediately according to the treatments described for each group. After 3 h of post-training sessions, one old object used during the training session was replaced by a novel object and the rat was left to explore the objects for 2 min. The time spent with each object was recorded and evaluated using ANY-maze software version 7.34 (Stoelting Co, Wood Dale, IL, USA). Both objects presented during the test session were different in texture, color, and size. The open field box was cleaned with 70% ethanol between runs to minimize scent trails. The recognition index was computed using the formula [TB/(TA + TB) * 100], where TA and TB are time spent exploring familiar object A and novel object B, respectively [46]. Exploration of an object was deemed when a rat sniffed or touched the object with its nose and/or forepaws.

#### 4.4.2. MWM Assay

MWM is a widely used tool for assessing spatial learning and memory [47]. The water maze was a stainless steel circular pool, 150 cm in diameter and 60 cm high, filled with 20 ± 1 °C water to a depth of 30 cm. The maze was topographically divided into four equal quadrants with defined release points at each quadrant marked as N, E, S, and W. A hidden circular plexiglas platform (10 cm in diameter) was located in the center of the southeast quadrant and submerged 1.5 cm below the water level, thereby requiring the rats to find the platform based on their memory function. During the training (T1 to T4), the rats received pre-treatment and treatment as previously described. Then, the rats were trained to find the hidden platform during 120 seg. If the rat did not find the platform, it was placed by the experimenter on the platform and kept there for 30 s. Afterwards, the rat was removed from the platform to be gently dried and to immediately receive saline solution or scopolamine, according to the treatments described for each group. The latency and pathway to search the hidden platform were recorded and used to evaluate the ability of learning and memory. Latency to find the hidden platform during T1 to T4, and the time exploring the quadrant where the platform was located versus the opposite quadrant on trial day when the hidden platform was removed, were examined.

### 4.5. Effect of Blockading H1R and HR2 on the Effects on Memory Produced by DHE in the Scopolamine-Induced Amnesia Model

To determine the contribution of increased histamine levels in the brain on the effects on memory produced by DHE, pre-treatment with pyrilamine (an H1R antagonist) or famotidine (an H2R antagonist) was evaluated. For this purpose, twenty Wistar rats were randomized into 2 groups (n = 10) to undergo behavioral assays employing the NOR paradigm (days 1 and 2) and MWM (days 3 to 7). Pre-treatments and treatments were administered for each group as follows: DHE + Pyrilamine group: receive pre-treatment with pyrilamine 20 mg/kg i.p. (30 min before behavioral training), treatment with DHE 1 mg/kg i.p. (20 min before behavioral training), and scopolamine 2 mg/kg immediately after the training. DHE + Famotidine group: receive pre-treatment with famotidine 10 mg/kg i.p. (30 min before behavioral training), treatment with DHE 1 mg/kg i.p. (20 min before behavioral training), and scopolamine 2 mg/kg i.p. immediately after the training. Behavioral assays for NOR and MWM were performed as described previously in Section 4.4.1 and Section 4.4.2.

### 4.6. Statistical Analysis

Data obtained from all studies were expressed as mean ± SEM. Statistical analysis was performed using paired Student *t*-tests or one-way analysis of variance (ANOVA), using Tukey’s test as a post-hoc test. *p*-values of * *p* < 0.05 were considered statistically significant. 

## 5. Conclusions

The present work demonstrated that dihydroergotamine improves memory in the scopolamine-induced amnesia model through increasing histamine levels in the hippocampus by its activity as an HNMT inhibitor. 

## Data Availability

The data reported in this study are available upon request to dra.hernandez.ipn@gmail.com.
